# Aortic Regurgitation Quantification using Cardiac Magnetic Resonance. Is there a best imaging plane for flow quantification? A single center clinical trial

**DOI:** 10.1186/1532-429X-18-S1-P340

**Published:** 2016-01-27

**Authors:** Henrique S Trad, Ana Marta A Gali, Marcel Koenigkam Santos, Maria Fernanda Braggion-Santos, Gustavo J Volpe, Benedito C Maciel, Andre Schmidt

**Affiliations:** 1 Cardiology, HCFMRP, Ribeirao Preto, Brazil; 2 Radiology, CCIFM, HCFMRP, Ribeirao Preto, Brazil

## Background

The precise quantification of aortic regurgitation (AR) is central to surgical planning. Some authors suggest early intervention in asymptomatic patients with severe AR. With the advent of transcatheter aortic valve implantation (TAVI), the identification and precise quantification of paravalvular regurgitation is also paramount. In routine clinical practice, the imaging plane used for flow analysis by phase contrast (PC) technique is the sinotubular junction (STJ) in the ascending aorta. However, this plane is not suitable in cases with TAVI. The other two used planes are the ascending aorta (AAo) and the left ventricle outflow tract (LVOT), but these are said to respectively, overestimate and underestimate the measurements.

**Objective:** to compare the volumes of three different planes of measurement in the AAo, JST and LVOT, in patients with different degrees of AR.

## Methods

Patients with isolated AR underwent cardiac magnetic resonance (CMR) with PC through-plane flow quantification in the three above mentioned planes. Pearson correlations and paired t-Student tests were performed with the different planes measures, with a significance level of 5 %.

## Results

95 patients with isolated AR were evaluated, 8 of them with bicuspid aortic valves. Significant correlation (p < 0.0001) was present between forward and regurgitant flow, and the regurgitant fraction throughout the series, with r values ranging from 0.83 to 0.96. In the evaluation of volumes, a significant difference was present between forward flow in the AAo and STJ (111 ± 42 ml vs. 108 ± 43 ml, p = 0.006) although with little clinical relevance. No significant difference between planes was present in any volumes or regurgitant fraction in patients with bicuspid aortic valves, when separately analyzed.

## Conclusions

In AR patients, flow quantification similarities suggests that, when the usual plane of analysis is impaired, reliable measures can be obtained in different planes in ascending aorta and LVOT.

